# Frontal EEG alpha activity and obsessive-compulsive behaviors in non-clinical young adults: a pilot study

**DOI:** 10.3389/fpsyg.2015.01480

**Published:** 2015-09-29

**Authors:** Michael Wong, Erik Z. Woody, Louis A. Schmidt, Michael Van Ameringen, Noam Soreni, Henry Szechtman

**Affiliations:** ^1^Department of Psychiatry and Behavioural Neurosciences, McMaster University, Hamilton, ON, Canada; ^2^Department of Psychology, University of Waterloo, Waterloo, ON, Canada; ^3^Department of Psychology, Neuroscience, and Behaviour, McMaster University, Hamilton, ON, Canada

**Keywords:** obsessive-compulsive disorder, security motivation, frontal hypoactivity, electroencephalography, anxiety

## Abstract

Previous studies have shown that the resting electroencephalogram (EEG) alpha patterns of non-clinical participants who score high on measures of negative affect, such as depression and shyness, are different from those who score low. However, we know relatively little about patterns of resting EEG alpha patterns in a non-clinical sample of individuals with high levels of obsessive-compulsive behaviors indicative of obsessive-compulsive disorder (OCD). Here we measured resting EEG alpha activity in frontal and parietal regions of non-clinical participants who scored high and low on the Padua-R, a measure of the severity of OCD-related behaviors. We found that participants who scored high on the Padua-R exhibited decreased overall activity in frontal regions relative to individuals who scored low on the measure. We speculate that frontal hypoactivity may be a possible marker and/or index of risk for OCD.

## Introduction

Obsessive-compulsive disorder (OCD) is a psychiatric condition that has an estimated lifetime prevalence of about 1–2% (Rasmussen and Eisen, 1991; Kessler et al., 2005). Patients with OCD feel the need to perform repetitive behaviors (compulsions), often associated with feelings of anxiety and recurring and intrusive thoughts (obsessions). For example, an individual may feel compelled to wash his or her hands for prolonged periods of time together with experiencing feelings of anxiety and thoughts of getting sick.

Studies have shown that resting electroencephalogram (EEG) alpha patterns distinguish patients who have affective disorders from healthy control participants. For example, greater relative right (vs. left) resting frontal EEG activity has been observed in patients who have been clinically diagnosed with panic disorder (Wiedemann et al., 1999), social anxiety disorder (Davidson et al., 2000; Moscovitch et al., 2011), and depression (Henriques and Davidson, 1991). Interestingly, this pattern of frontal asymmetry has also been observed in non-clinical participants who self-report high levels of negative affect, such as depression (Schaffer et al., 1983) and shyness (Schmidt, 1999). Accordingly, the pattern of frontal EEG alpha activity at rest might serve as an index of risk and/or predisposition for affective disorders prior to onset of disorder. To our knowledge, however, individual differences in resting frontal EEG activity have not been investigated in a non-clinical sample of adults exhibiting high OCD-related behaviors. In the present study, we examined whether young adults who self-reported high vs. low levels of OCD-related behaviors, but who had not been clinically diagnosed with OCD, would exhibit differences in resting frontal EEG alpha activity.

Several studies have recorded regional EEG alpha activity in individuals with clinically diagnosed OCD, but these results are mixed. For example, some studies have suggested that patients with OCD have greater relative right resting frontal activity than healthy control participants (Kuskowski et al., 1993; Ischebeck et al., 2014), but other studies found no hemispheric differences between the two participant groups (Karadag et al., 2003; Bucci et al., 2004). Additionally, frontal dysfunction in patients with OCD has been suggested (see Khanna, 1988; Pogarell et al., 2006), but the results of these studies are also mixed. For example, some studies have suggested that patients with OCD have decreased activity in frontal regions (Desarkar et al., 2007), but other studies have reported increased activity in frontal regions (Karadag et al., 2003; Bucci et al., 2004).

Here we sought to clarify these inconsistent findings by comparing patterns of resting frontal EEG activity in a non-clinical sample of young adults who scored high and low on the Padua Inventory (Padua-R; Burns et al., 1996), a self-report measure of OCD-related behaviors. We recorded left and right frontal and parietal EEG alpha activity at rest. Our results revealed no hemispheric differences between the two participant groups, but lower frontal activity in participants who scored high on the Padua-R. Our finding of frontal hypoactivity possibly reflects a problem of cognitive inhibitory control, and suggests frontal brain dysfunctions may serve as a marker and/or premorbid brain correlate for OCD.

## Materials and Methods

### Participants

Twenty-nine young adult participants (mean age: 17.8 years, range: 16–18; 23 female, 6 male), who have not been clinically diagnosed with OCD, participated in the study. Twenty-four participants were recruited from the Department of Psychology, Neuroscience, and Behaviour undergraduate participant pool; and five participants were recruited from a child database, which contains the names of parents and children recruited at birth for later studies. None of the participants reported they were clinically diagnosed with a psychiatric condition or were on any medication; and two participants indicated they smoked cigarettes occasionally. All experimental procedures were approved by the Hamilton Integrated Research Ethics Board.

### Self-Report Measure

We administered the Padua-R (Burns et al., 1996), a 39-item self-report measure of OCD symptom severity, to our participants. Items in this inventory are scored on a 5-point scale, from 0 (not at all) to 4 (very much). Example items include “*I wash my hands more often and longer than necessary*” and “*Sometimes I am not sure I have done things which in fact I know I have done*.” Previous research has demonstrated that this inventory has high reliability and validity (Burns et al., 1996).

### Participant Grouping

We created two groups, based on their score on the Padua-R. Those participants who self-reported relatively higher (*n* = 14) than or equal to the median score of 48 formed the high OCD-symptom-severity group, and those who scored lower (*n* = 14) formed the low OCD-symptom-severity group (see Table 1, for the distribution of individual scores). This cutoff seemed appropriate because it divided our sample into two even groups of participants, and in previous studies the average Padua-R score for OCD patients was approximately 48 or higher (e.g., Marker et al., 2006; Hinds et al., 2012; Thomas et al., 2014).

**TABLE 1:** **Characteristics of participants in the low and high OCD-symptom-severity groups**.

**Low OCD-symptom-severity**				**High OCD-symptom-severity**			
**Participant**	**Age (year)**	**Sex**	**Padua-R**	**Participant**	**Age (year)**	**Sex**	**Padua-R**
1	18	F	5	15	18	F	81
2	18	F	13	16	18	F	49
3	18	F	14	17	18	F	70
4	18	F	41	18	17	F	89
5	18	F	31	19	18	F	66
6	16	F	22	20	18	F	90
7	18	F	22	21	18	F	102
8	18	F	27	22	18	M	80
9	18	F	25	23	18	M	79
10	18	M	33	24	18	M	52
11	18	M	17	25	18	F	57
12	16	M	39	26	18	F	73
13	18	F	42	27	18	F	62
14	18	F	47	28	17	F	86
Mean			27				74
SD			12.5				15.5

### Experimental Procedures

We collected a 2-min eyes-closed resting EEG recording from each participant. During this time, participants were seated comfortably in a chair and instructed to simply relax.

We recorded brain electrical activity using a lycra EEG stretch cap (Electro-Cap International Inc.) from the left and right frontal sites (F3, F4) and parietal sites (P3, P4), all referenced to the central vertex (Cz). Each electrode site (and a ground site) were gently abraded and filled with electrolyte gel (Nuprep gel and Electro-Gel). We ensured impedance for each recording site was below 5 kΩ. Regional EEG was recorded using a BIOPAC MP35 Data Acquisition System (BIOPAC Systems, Inc.) coupled with BSL PRO software (BIOPAC Systems, Inc.) for reduction. EEG alpha power (in μV) was derived from a fast-Fourier transform on all artifact free data. Given that EEG alpha power is inversely related to activity, lower power values reflect more activity (Laufs et al., 2003).

### Data Analysis

To examine whether there were regional EEG activity differences between the two OCD-symptom-severity groups, we performed a 2 (Region: frontal, parietal) × 2 (Hemisphere: right, left) × 2 (Group: high, low) × 2 (Sex: male, female) mixed-model ANOVA. The dependent measure was alpha (8–13 Hz) power during the 2-min resting period. This ANOVA and all *post hoc* tests were performed using SPSS Statistics v21 (IBM) for MacIntosh, with α-level of 0.05. Data from one female participant were missing given computer failure during data collection.

## Results and Discussion

**FIGURE 1:**
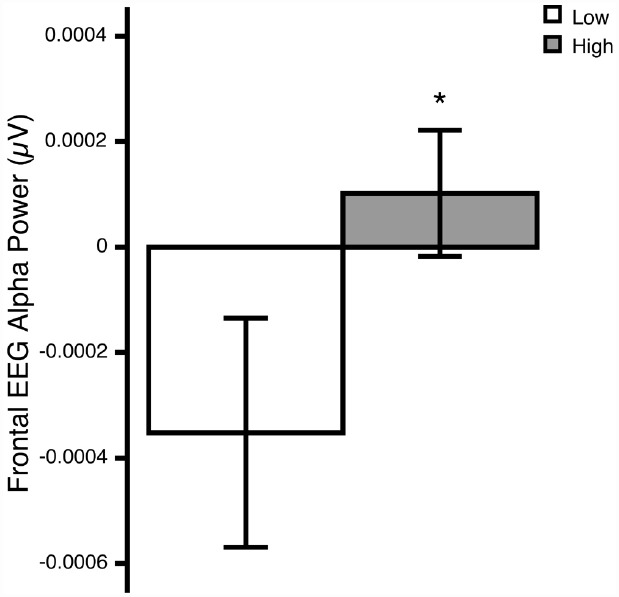
**Overall frontal EEG alpha (8–13 Hz) power at rest in young adults self-reporting low (white bars) and high (gray bars) OCD-related behaviors.** *p<0.05. Means ± 1 SE. Note: because alpha power is inversely related to activity, higher power reflects lower activity.

The ANOVA revealed a significant Region × Group interaction [*F*_(1,24)_ = 6.23, *p* = 0.020; ${\text{η}}_{p}^{2}$ = 0.21]. *Post hoc* comparisons revealed that, relative to the low OCD-symptom-severity group, the high OCD-symptom-severity group exhibited lower overall activity (i.e., higher power) in frontal regions (*p* = 0.039; Figure 1). There was no significant main effect of Hemisphere, nor was there a significant Hemisphere × Group interaction.

These preliminary results are consistent with several EEG studies of patients with OCD. For example, our finding of overall frontal hypoactivity is consistent with the results of Desarkar et al., 2007 (but see Karadag et al., 2003; Bucci et al., 2004). Additionally, our lack of finding frontal asymmetry differences is consistent with the results of Karadag et al. (2003) and Bucci et al., 2004 (but see Kuskowski et al., 1993; Ischebeck et al., 2014).

What does reduced overall frontal activity reflect in obsessive-compulsive behaviors? Our finding of frontal hypoactivity is interesting in light of the security-motivation hypothesis for OCD (Szechtman and Woody, 2004). According to this hypothesis, OCD is a defect in a special motivation system—the security motivation system (SMS)—that normally serves to protect the organism from potential harm. That is, when an organism encounters a potentially harmful stimulus, such as a possible contaminant, the SMS is activated to elicit a set of security-related behaviors, such as washing, to protect the organism; performance of these behaviors is proposed to terminate an activated security motivation. Within this framework, OCD can be conceptualized as either a problem of *starting*—in which a hyperactive SMS increases the intensity with which potentially harmful stimuli are perceived, or a problem of *stopping*—in which security-related behaviors fail to terminate the SMS (Woody and Szechtman, 2005).

Using respiratory sinus arrhythmia (RSA; a psychophysiological measure of peripheral nervous system activity) as an index of security motivation, our group (Hinds et al., 2012) recently found support for the latter: OCD is a problem of stopping. The results showed that immediately after contact with a potentially contaminated stimulus (see also Hinds et al., 2010), OCD patients and control participants did not differ in the level of security motivation activation, suggesting that patients with OCD did not perceive the stimulus with greater intensity than control participants did. However, there was a difference between the two participants groups in the length of time required to wash the hands to return security motivation to baseline—whereas healthy control participants required only a short 30-s wash period, OCD patients required a time unlimited wash period during which they were able to wash their hands for as long as they wished. That is, the security-related behavior of washing appeared to be less able to terminate the SMS in patients with OCD than in healthy control participants.

The results of the present pilot study possibly provide additional support for the stopping hypothesis. The frontal lobe has been implicated in cognitive regulation and inhibitory control. Additionally, frontal hypoactivity has been observed in a number of impulse disorders, including, for example, attention deficit hyperactivity disorder (Dickstein et al., 2006), substance-related disorders (Goldstein and Volkow, 2011), and borderline personality disorder (Salavert et al., 2011). Thus, the present results suggest that OCD may be more like impulse disorders (with regard to frontal hypoactivity) than like anxiety disorders (with regard to overall frontal hyperactivity and/or right-frontal hyperactivity). This idea is consistent with the new categorization of OCD in the DSM-5, where OCD has been moved out of the anxiety disorders and into a group of impulse-like disorders (American Psychiatric Association, 2013).

The decreased activity we observed in frontal regions of the high OCD-symptom-severity group may suggest these participants require more intense performance to generate the negative feedback signal to terminate SMS activity, and hence frontal hypoactivity may serve as a marker and/or index of risk for OCD. Although our participants have not been clinically diagnosed with OCD, the Padua-R scores of the high OCD-symptom-severity group fell within the range typically reported for an OCD population; therefore, this sample of participants may be more similar to that of an OCD population than a healthy population. Future studies should therefore be conducted using a larger sample of participants who have been more rigorously screened for the presence or absence of OCD to ensure the reliability and generalizability of the present pilot data. Additionally, given the heterogeneous nature of OCD symptoms, future studies should also aim to investigate whether EEG dysfunctions in subsyndromal OCD are symptom specific as they are in clinical cases of OCD (Pogarell et al., 2006).

### Conflict of Interest Statement

The authors declare that the research was conducted in the absence of any commercial or financial relationships that could be construed as a potential conflict of interest.
